# *Corryocactus brevistylus* (K. Schum. ex Vaupel) Britton & Rose (Cactaceae): Antioxidant, Gastroprotective Effects, and Metabolomic Profiling by Ultrahigh-Pressure Liquid Chromatography and Electrospray High Resolution Orbitrap Tandem Mass Spectrometry

**DOI:** 10.3389/fphar.2020.00417

**Published:** 2020-04-08

**Authors:** Carlos Areche, Marco Hernandez, Teresa Cano, Juana Ticona, Carmen Cortes, Mario Simirgiotis, Fátima Caceres, Jorge Borquez, Javier Echeverría, Beatriz Sepulveda

**Affiliations:** ^1^Departamento de Química, Facultad de Ciencias, Universidad de Chile, Santiago, Chile; ^2^Departamento de Química, Facultad de Ciencias Naturales y Formales, Universidad Nacional de San Agustín, Arequipa, Perú; ^3^Instituto de Farmacia, Facultad de Ciencias, Universidad Austral de Chile, Valdivia, Chile; ^4^Laboratorio de Botánica, Departamento de Biología, Facultad de Ciencias Biológicas, Universidad Nacional de San Agustín, Arequipa, Perú; ^5^Departamento de Química, Facultad de Ciencias Básicas, Universidad de Antofagasta, Antofagasta, Chile; ^6^Departamento de Ciencias del Ambiente, Facultad de Química y Biología, Universidad de Santiago de Chile, Santiago, Chile; ^7^Departamento de Ciencias Químicas, Universidad Andrés Bello, Viña del Mar, Chile

**Keywords:** Cactaceae, *Corryocactus brevistylus*, sancayo, flavonoids, phenolics compounds, gastroprotective, gastric ulcer

## Abstract

*Corryocactus brevistylus* (K. Schum. ex Vaupel) Britton & Rose (Cactaceae) is a shrubby or often arborescent cactus popularly known as “sancayo” and produce an edible fruit known as “Sanky” which is consumed in Arequipa-Perú. The purpose of this study was to report the gastroprotective activity and relate this activity to the antioxidant capacity and presence of phenolic compounds for the first time. A metabolomic profiling based on Ultrahigh-pressure liquid chromatography and electrospray high resolution mass spectrometry, and the antioxidant activities (DPPH, ABTS, and FRAP), ascorbic acid content, total phenolics and flavonoids contents, and the mode of gastroprotective action of the Sanky fruit including the involvement of prostaglandins, nitric oxide, and sulfhydryl compounds is reported. Thirty-eight compounds were detected in the ethanolic extract including 12 organic acids, nine hydroxycinnamic acids, three isoamericanol derivatives, six flavonoids, five fatty acids, and two sterols. The results of the biological tests showed that the ethanolic extract had antioxidant capacity and gastroprotective activity on the model of HCl/EtOH-induced gastric lesions in mice (at 10, 25, 50, and 100 mg/kg). The effect elicited by the extract at 50 mg/kg was reversed by indometacin and *N*-ethylmaleimide but not by N^G^-nitro-L-arginine methyl ester suggesting that sulfhydryl groups and prostaglandins are involved in the mode of gastroprotective action. In conclusion, our study proves that *C. brevistylus* pears have some gastroprotective and antioxidant capacities and consumption is recommended for the presence of several bioactive compounds.

## Introduction

Today, fruits are considered the healthiest food in human health. Several native edible plants have played a significant role in all geographical regions of the world in human history and many local fruits are commercially available as juice, jam, or manufactured products (cookies, ice cream, yogurt, or dried fruits) due to the endemic knowledge of their health beneficial properties supported by their content of fiber, vitamins, and minerals plus bioactive compounds (Dreher, 2018; Pepin et al., 2019). Indeed, there is growing interest on the identification, composition, and nutritional properties of endemic edible fruits by the population and the scientific community (Yeung et al., 2018). In this sense, many foods have been studied and the technological progress each day finds new biological applications on fruits and fruit products. Among endemic South American edible fruits, we can find *Opuntia* prickly pear (Aruwa et al., 2019; Smeriglio et al., 2019), chayote pear (Díaz-de-Cerio et al., 2019), cherimola pulp (Albuquerque et al., 2016), strawberry (Baby et al., 2018), maqui (Rivera-Tovar et al., 2019), and murta (Rivera-Tovar et al., 2019) among others.

**Figure 1:**
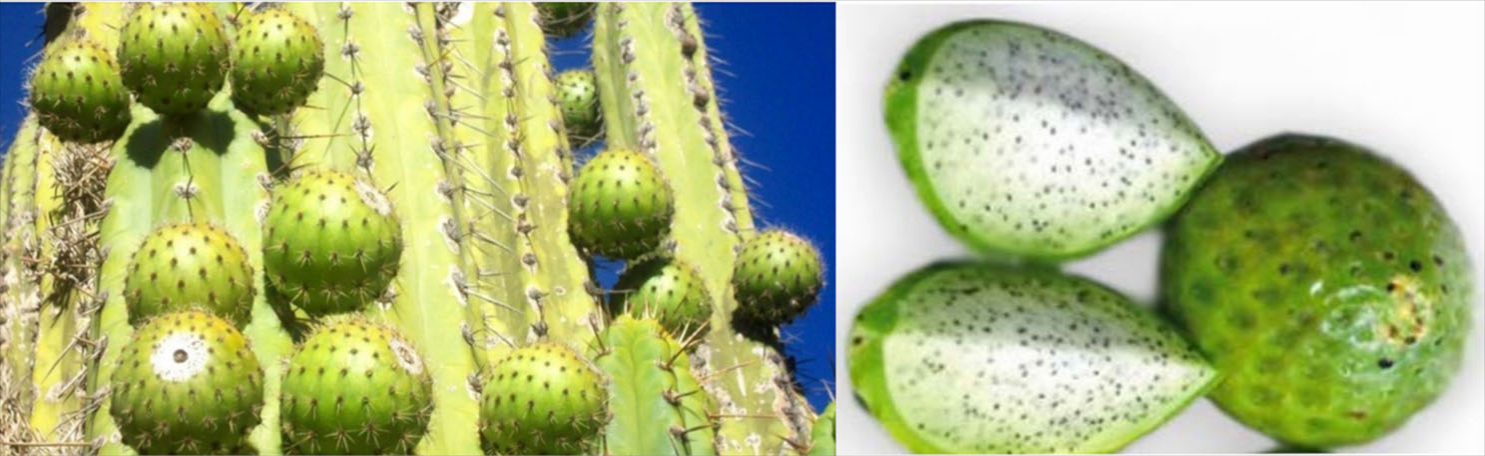
*Corryocactus brevistylus* fruits.

The Cactaceae family in Perú comprises 43 genera including 250 species where almost 80% of these species are endemic (Ostolaza Nano, 2014). The tribe Notocacteae include 10 genera, and only four genera have been found in Perú including Corryocactus, Eulychnia, Islaya, and Neowerdermannia. In Perú, *Corryocactus brevistylus* (K. Schum. ex Vaupel) Britton & Rose (Cactaceae, **Figure 1**) is a shrubby or often arborescent cactus popularly known as “*sancayo*”, which it is distributed in South America from Yura in Arequipa-Perú to Iquique in Northern Chile. According to Ostolaza; 2014, *C. brevistylus* is a source of edible fruits known as “Sanky” which are consumed and marketed by peasants in Arequipa for its high ascorbic acid content (Ostolaza Nano, 2014). It is considered effective to prevent liver diseases and gastritis. According to the information provided by the residents of Arequipa-Perú, the peel of the fruit is used to strengthen the scalp and it was never seriously considered as a crop for a rational exploitation [neglected and underutilized species (NUS)]. In Chile, this cactus is very abundant in the Andes of Iquique (Quebrada de Chusmiza, Mamiña, and Chiapa) and the Chapiquiña-Belén, where it is called, *cardon verde, guacalla*, and *tacaysiña*. Its edible fruit is acidic and receives several vernacular names (*maksa*, *kontumela*, *kontumila*, *kontoksa*, *kontuksa*, *romba*, or *rumba*) and has various medicinal properties. It is consumed in the morning, with empty stomach, for gallbladder problems, stomach pain, liver, kidney stones, and as a laxative (Villagrán and Castro, 2003; Echeverría et al., 2020). *C. brevistylus* grows at about 4,000 meters above the sea level and their stems reach up to 2–5 m tall, forming large groups of dark green to light yellowish-green, with thorns of up to 24 cm long. Its flowers are yellow between 5–6 cm long x 10 cm wide. *Corryocactus brevistylus* fruit is green-yellowish, juicy, 12 cm long with abundant thorns and are found in saline soils and having a neutral acid flavor (Rispoli and Chipana, 2018). This edible fruit was used since ancient times by the Incas as a natural energizer to support their long Andean trips (Ostolaza Nano, 2014). Almost nothing is known on the chemistry and biological effect of Sanky (*C. brevistylus* fruits). Therefore, no information on the presence of their metabolites has been informed to date.

High performance liquid chromatography (HPLC) or ultrahigh-pressure liquid chromatography (UHPLC) coupled to high resolution-mass spectrometry is today a fundamental tool for the identification of phenolic compounds in edible fruits. Among the instruments offered in the market, the Q-Exactive Focus uses a very rapid high-resolution mass spectrometer for the detection of organic molecules equipped with an orbital trap, a quadrupole (Q) plus and an HCD cell (High-Resolution Collision), producing parent ions and daughter fragments at high-resolution and good sensitivity. The hyphenated UHPLC-MS approach is suitable for the analysis of small organic compounds up to 2,000 Daltons, including fruit pigments, organic acids, flavonoids, pesticides, toxins, and terpenes (Perez et al., 2016).

In the course of our investigation on NUS Andean plants with potentiality as new crops, we now report the gastroprotective and antioxidant capacities, phenolic and flavonoid contents, and phenolic profiling based on Ultra HPLC electrospray high resolution mass spectrometry (UHPLC/ESI/HR-MS) plus the mode of gastroprotective action of *C. brevistylus* fruits including the mechanism of action [involvement of nitric oxide (NO), prostaglandins (PGs), and sulfhydryls (SHs)].

## Materials and Methods

### Plant Material

Ripe fruits from *C. brevistylus* (K. Schumann ex Vaupel) Britton et Rose were collected by hands in January 2016, in Chiguata (16˚ 24′ 09″ S, 71˚ 24′ 56″ W), Arequipa (Perú). The fruits were transported to the laboratory under refrigeration until processing. The species was identified by Dr. Fatima Caceres, Biology Department, San Agustin National University, Arequipa, Perú. A voucher specimen (N° CB-15012016) was deposited at the Herbarium of the San Agustin National University.

### Fruit Processing

*Corryocactus brevistylus* fruits were divided into epicarp (peel) and pulp. The pulp was frozen (465 g) and then lyophilized obtaining 50 g of dried fruit powder. This powder (40 g) was extracted three times with ethanol (500 mL each time) at room temperature under sonication (15 min each). The solutions were filtered, combined and evaporated under reduced pressure below 40°C to obtain 8 g of crude ethanolic extract (CEX).

### Identification of Phenolic Compounds by UHPLC-ESI-HR-MS/MS

Equipment: A Thermo Scientific Dionex Ultimate 3000 UHPLC system operated by Chromeleon 7.2 Software (Thermo Fisher Scientific, Bremen, Germany) hyphenated with a Thermo high resolution Q Exactive focus mass spectrometer (Thermo, Bremen, Germany) were employed for analysis. Nitrogen obtained from a generator (Ciantecnologica, Seville, Spain) was used as both the collision and damping gas. All calibration and equipment parameters were set as reported (Simirgiotis et al., 2016).

LC parameters: The column used was a UHPLC C18 column (Acclaim, 150 mm × 4.6 mm ID, 2.5 µm, Restek Corporation, Bellefonte PA, USA) operated at 25 °C. The detection was set at 320, 254, 280, and 440 nm, and 800–200 nm in the PDA was recorded. Mobile phases were 1% formic aqueous solution (A) and acetonitrile with 1% formic aqueous solution (B). The gradient program was: (0.00, min, 5% B); (5.00 min, 5% B); (10.00 min, 30% B); (15.00 min, 30% B); (20.00 min, 70% B); (25.00 min, 70% B); (35.00 min, 5%B); and 12 minutes before each injection for equilibration. The flow rate was set at 1.00 mL min^−1^, and the injection volume was 10 µL.

MS parameters: The HESI II and other parameters were optimized as previously reported (Simirgiotis et al., 2016).

### Chemicals

Ultra-pure water [< 5 µg/L TOC, (total organic carbon)] was obtained from a water purification system Arium 126 61316-RO, plus an Arium 611 UV unit (Sartorius, Göttingen, Germany). Methanol (HPLC grade), ethanol, formalin, and formic acid (puriss. p.a. for mass spectrometry) from J. T. Baker (Phillipsburg, NJ, USA) were obtained. Folin-Ciocalteu reagent, sodium nitrite, trolox, sulfuric acid, copper sulfate, and aluminum chloride were from Merck (Santiago, Chile). DPPH reagent, (2,2-diphenyl-1-picrylhydrazyl), lansoprazole, ferric chloride hexahydrate, 2,4,6-tris(2-pyridyl)-s-triazine, *N*-ethylmaleimide (NEM), Indomethacin (IND), *N*-nitro-L-arginine methyl ester (L-NAME), oxalic acid, trolox, ascorbic acid, quercetin, gallic acid, DMSO, and HPLC standards (rutin, rhamnetin, and isorhamnetin, all standards with purity higher than 95% by HPLC) were purchased from Sigma-Aldrich Chem. Co. (St Louis, MO, USA) or Extrasynthèse (Genay, France).

### Mice

Animals were bought from the Institute of Public Health of Chile, Santiago. Swiss albino mice in metabolism cages were fasted for 24 h. Mice were weighed (30 ± 3 g) before the experiments. Mice were fed using certified diet (Champion) with free access to water under standard conditions (50% relative humidity, 12 h dark-light period, and 22°C room temperature). These protocols were approved by the Animal Use and Care Committee of the Universidad de Chile that follows the recommendations of the Canadian Council on Animal Care (Olfert et al., 1993). Such certificate was approved in July 2014 by Dr. Nicolas Giuliani with number AUCC-02072010.

### HCl/Ethanol-Induced Lesions

The gastroprotective effect of the CEX was evaluated in the HCl/EtOH-induced lesion model (Matsuda et al., 2002; Parra et al., 2015). Mice were randomly placed into groups of seven animals each and fasted for 24 h with free water access prior to the experiments. Fifty min after oral administration of CEX (10, 25, 50, and 100 mg/kg), lansoprazole (30 mg/kg) or 10% Acacia gum (vehicle), all groups were orally dispensed with 0.2 mL of a solution containing 0.3 M HCl/60% ethanol (HCl/EtOH) for gastric lesion induction. The CEX was administered at 50 mg/kg in a second experiment to evaluate its possible mode of gastroprotective action using carbenoxolone (100 mg/kg) as a positive control. Mice were sacrificed 60 min after the administration of HCl/EtOH, and the stomachs were excised and inflated by injection of saline solution (1 mL). The ulcerated stomachs were fixed in 5% formalin for 30 min and opened along the greater curvature. Gastric damage was observed in the gastric mucosa as elongated black-red lines, parallel to the long axis of the stomach. The length (mm) of each lesion was measured, and the lesion index was expressed as the sum of the length of all lesions.

### HCl/Ethanol-Induced Gastric Lesions in IND-, NEM-, and L-NAME-Pretreated Mice

To study the involvement of endogenous prostaglandins, sulfhydryl compounds and endogenous nitric oxide in the gastroprotective activity of CEX, IND s.c. (30 mg/kg, an inhibitor of the prostaglandin synthesis was dissolved in 5% NaHCO_3_); NEM s.c. (10 mg/kg, an SH blocker), and L-NAME i.p. (70 mg/kg, an inhibitor of NO synthase) were injected 30 min before administration of CEX or vehicle (Matsuda et al., 2002; Parra et al., 2015). Fifty min after oral administration of CEX (50 mg/kg) or vehicle, all groups were orally treated with 0.3 M HCl/60% ethanol solution (0.2 mL) for gastric lesion induction. Mice were sacrificed 60 min after the administration of HCl/EtOH, and the stomachs were opened and inflated by injection of saline (1 mL). The length of gastric lesions was measured as described above.

### Antioxidant Capacity and Content of Phenolic Compounds

#### Polyphenol and Flavonoid Contents

The assay of total phenolic compounds (TPC) was done based on Ramirez et al. (2014). Some 12 μL of extract, 168 μL of the 1% Folin-Ciocalteu reagent (Merck) were added to a well of a microplate reader. The mixture was left to react for 5 min, then 120 μL of 10% Na_2_CO_3_ was added. The mixture was incubated at room temperature for 30 min in darkness. Absorbance was then measured at 765 nm using an UV-visible multiplate reader (Synergy HTX, USA). The content regarding phenolic compounds was then expressed as gallic acid micromoles per gram of dry weight (μmol GAE/g extract). The AlCl_3_ method was used for the determination of the total content of flavonoids. For this test, 30 μL of the sample (2 mg/mL) was added to 159 µL of 5% NaNO_2_. After 5 min of rest, 18 μL of 10% AlCl_3_ was added to the mixture. At the sixth minute, 18 μL of 1 M NaOH was completed and the absorbance measured at 510 nm using an UV-visible multiplate reader (Synergy HTX, USA). Flavonoid content (TFC) was calculated using a quercetin standard calibration curve (25–150 ppm). Results were expressed as quercetin micromoles per gram of dry sample (μmol Q/g dry weight).

#### Determination of Ascorbic Acid Content

This assay was performed according to method published previously (Brito et al., 2014). The sample (0.3 g of pulp) was extracted with freshly prepared 0.5% oxalic acid, centrifuged at 2500 g for 10 min at 4°C, and filtered (PTFE, 0.2 µm). Then, the extracted sample and ascorbic acid (1.2 mL) were added to a capped test tube and then 0.4 mL of 2,4-dinitrophenylhydrazine: thiourea: copper sulfate (DTCS, 20:1:1 v/v/v) reagent was added and incubated in a water bath 3 h at 37°C. Then, 2.0 mL of cold sulfuric acid (12 M) was added while mixing. The absorbance was measured immediately against the prepared blank at 520 nm. The standard curve was done with L-ascorbic acid (1–25 mg/L; r^2^ = 0.9995) and the results were expressed as mg of ascorbic acid per 100 g of fresh fruit.

#### DPPH Test

The capturing capacity of the DPPH• radical was measured by the decolorization method (Brito et al., 2014; Ramirez et al., 2014). Briefly, 9 μL of CEX extract, (2 mg/mL), plus 341 μL of methanol DPPH solution (400 μM) were adjusted with methanol to an absorbance of 1.10 ± 0.02 at 517 nm. The mixture was homogenized, allowed to react in the dark at room temperature for 20 min, after which time absorbance was measured at 517 nm. The percentage of discoloration of the DPPH radical was obtained by measuring the change in absorbance at 517 nm, the values obtained converted to percent inhibition of the DPPH moiety. A curve was performed with different dilutions of the extract, (5–50 mg/L; r^2^ = 0.9998) and the results were expressed as half maximal inhibitory concentration (IC_50_) in µg/mL.

#### ABTS•+ Radical Scavenging Assay

The capturing capacity of the ABTS•+ radical was performed as reported previously (Brito et al., 2014; Ramirez et al., 2014). The ABTS•+ radical is generated by the oxidation of ABTS with potassium persulfate after 16 h of incubation at room temperature in the dark. Briefly, 27 μL of the CEX extract to be measured, (2 mg/mL), was added to 273 μL of the previously prepared ABTS•+ solution (adjusted with 80% methanol to obtain an absorbance of 0.70 ± 0.02 at 734 nm), to the well of the microplate (Synergy HTX, Biotek USA) and subsequently allowed to react in darkness at room temperature for 6 min. The absorbance was then measured at 765 nm and the values obtained converted to % inhibition of the ABTS•+ radical and the results were expressed as IC_50_ in μg/mL.

#### Ferric Reducing Antioxidant Power Test (FRAP)

For the FRAP assay, the methodology was performed as published with slight modifications (Brito et al., 2014; Ramirez et al., 2014). Briefly, to 10 μL of the dissolved extract (2 mg/mL), 290 μL of the FRAP solution was added and mixed in the well of the microplate, allowed to react in the dark at room temperature for 5 min. The absorbance measurement of the colored Fe-TPTZ complex was done at 595 nm. Absorbance data were replaced in the Trolox standard curve equation (μmol/L). The results were obtained as equivalents of Trolox (TE), in Trolox micromoles per gram of dry weight (μmol Trolox/g dry weight).

### Statistical Analysis

Results were expressed as the mean ± S.D. In all experiments, statistical differences between treatments and controls were determined by one-way analysis of variance (ANOVA) followed by Dunnett’s test. The level of significance was set at P < 0.01. All statistical analyses were performed using the software GraphPad Prism 6 for Windows.

## Results

### Identification of Phenolic Compounds

**Figure 2:**
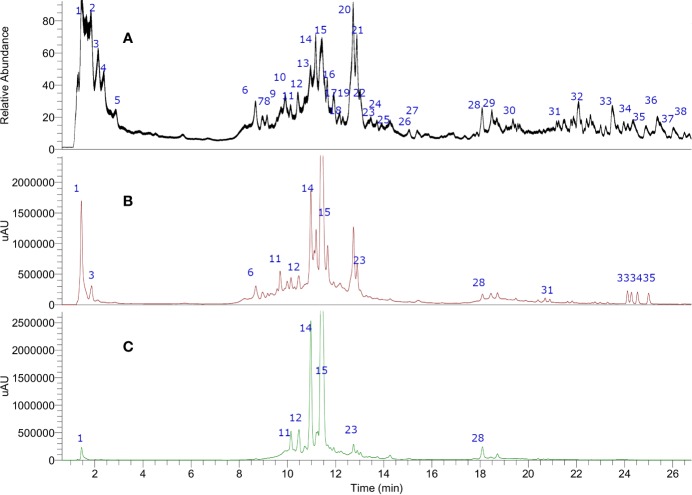
Ultrahigh-pressure liquid chromatography (UHPLC) chromatogram of **(A)** total ion current **(B)** UV at 254 nm and **(C)** 330 nm of *Corryocactus brevistylus* edible fruit.

In this study, 38 compounds were detected by UHPLC/PDA and electrospray high resolution orbitrap tandem mass spectrometry (UHPLC-ESI-HR-MS) in CEX of *Corryocactus brevistylus* fruits (**Figure 2**). Those compounds contained in the CEX were detected on a UHPLC and identified or tentatively characterized based on UV spectra and full HR-MS spectra plus diagnostic fragmentations (**Table 1**) in electrospray negative mode, since negative mode is the best mode of ionization for phenolic compounds. (The phenols loose easily a proton and the charge are delocalized, so it is the normal way of ionization for those compounds). A total of 37 peaks including 13 organic acids (peaks 1–8, 10–11, and 19–21), nine hydroxycinnamic acids (peaks 9, 12–14, 16, 19, and 22–24), three isoamericanol derivatives (25–27), six flavonoids (peaks 15, 17–18, 28–30), five fatty acids (peaks 31–33 and 35–36), and two sterols (peak 34 and 38) were identified taking into account their elution order and comparing their MS data with respect to plant metabolites reported in the literature and spiking experiments using standards (Gómez-Romero et al., 2011; Simirgiotis, 2013; Brito et al., 2014; Ramirez et al., 2014; Makita et al., 2016; Simirgiotis et al., 2016; Su et al., 2016; Guerrero-Castillo et al., 2019). Peak 37 is considered unknown.

**Table 1:** Identification of compounds in *Corryocactus brevistylus* fruits by UHPLC-PDA-ESI-HRMS/MS.

Peaks	UV Max (nm)	Tentative identification	[M-H]^−^	RT (min)	Theoretical Mass (*m/z*)	Measured Mass (*m/z*)	Accuracy (ppm)	MS^n^ ions (*m/z*)
**1**	–	Malic acid	C_4_H_5_O_5_^−^	1.46	133.0132	133.0136	2.1	115.0030
**2**	–	(iso) citric acid	C_6_H_7_O_7_^−^	1.82	191.0197	191.0190	3.7	173.0080; 111.0078
**3**	–	Hydroxyglutaric acid	C_5_H_8_O_5_^−^	2.10	147.0288	147.0291	2.0	129.0184; 111.0078
**4**	–	Hydroxyglutaric acid isomer	C_5_H_8_O_5_^−^	2.39	147.0288	147.0291	2.0	129.0184; 111.0076
**5**	–	Homo (iso) citric acid	C_7_H_9_O_7_^−^	2.86	205.0342	205.0349	3.4	187.0245; 161.0451, 111.0078
**6**	280, 211	Hydroxybenzoic acid	C_7_H_6_O_3_^−^	8.66	137.0244	137.0237	5.1	93.1205
**7**	–	Dehydroshikimic acid	C_7_H_11_O_5_^−^	8.96	175.0612	175.0604	4.6	123.0440
**8**	211	Ascorbic acid	C_6_H_7_O_6_^−^	9.14	175.0248	175.0243	2.9	115.0071; 87.00942
**9**	213, 287, 326	Caffeoyl-*O*-hexoside	C_15_H_17_O_9_^−^	9.33	341.0873	341.0878	1.5	179.0549; 163.0395
**10**	217, 288	Benzoyl aspartic acid derivative	C_11_H_10_O_5_N^−^	9.74	236.0553	236.0561	3.4	192.0660
**11**	217, 290, 323	Benzoyl aspartic acid derivative	C_11_H_10_O_5_N^−^	9.88	236.0553	236.0562	3.3	192.0660
**12**	221, 255, 342	Caffeoyl (iso) citric acid	C_15_H_13_O_10_^−^	10.14	353.0514	353.0513	0.3	191.0191; 173.0085 154.9978; 111.0078
**13**	221, 266, 331	Caffeoyl (iso) citric acid	C_15_H_13_O_10_^−^	10.42	353.0514	353.0513	0.3	191.0191; 173.0085 111.0078
**14**	221, 268, 318	Coumaroyl (iso) citric acid	C_15_H_13_O_9_^−^	10.68	337.0565	337.0564	0.4	163.0394; 154.9977 111.0078
**15**	254, 354	Rutin*	C_27_H_29_O_16_^−^	10.93	609.1461	609.1451	1.6	301.0345 179.0343; 151.0393
**16**	225, 288, 327	Coumaroyl (iso) citric acid	C_15_H_13_O_9_^−^	11.16	337.0565	337.0563	0.6	173.0086; 154.9977 111.0078
**17**	255, 350	Isorhamnetin-*O*-rutinose or Isorhamnetin-*O*-neohesperidose	C_28_H_31_O_16_^−^	11.42	623.1617	623.1607	1.5	315.0509, 301.0353
**18**	228, 290	Taxifolin	C_15_H_11_O_7_^−^	11.67	303.0510	303.0509	0.5	163.0394; 125.0235
**19**	227, 288, 327	Feruloyl (iso) citric acid	C_16_H_15_O_10_^−^	11.90	367.0670	367.0671	0.3	193.0506; 179.0344; 163.0395; 111.0079
**20**	223, 290, 321	Benzoyl aspartic acid derivative	C_13_H_12_O_5_N^−^	12.15	262.0553	262.0562	3.5	–
**21**	224	Azelaic acid	C_9_H_15_O_4_^−^	12.30	187.0964	187.0972	4.2	169.0859; 157.1228, 125.0964
**22**	225, 312	Methylcoumaroyl(iso)citric acid	C_16_H_15_O_9_^−^	12.72	351.0722	351.0720	0.5	173.0084; 163.0394 119.0494
**23**	226, 311	Methylcoumaroyl (iso) citric acid	C_16_H_15_O_9_^−^	12.89	351.0722	351.0719	0.4	163.0394;119.0494
**24**	225, 314	Methylferuloyl (iso) citric acid	C_17_H_17_O_10_^−^	13.01	381.0827	381.0826	0.3	193.0502; 111.0079
**25**	223, 283	Unknown (possibly isoamericanol B1, C1)	C_27_H_25_O_9_^−^	13.42	493.1493	493.1500	1.4	327.0873, 163.0394
**26**	223, 285, 328	Unknown (possibly isoamericanol fragment or derivative)	C_18_H_15_O_6_^−^	13.71	327.0863	327.0874	2.3	163.0394; 147.0444
**27**	195, 223, 283	Unknown (possibly isoamericanol B1 or its isomer C1, B1)	C_27_H_25_O_9_^−^	13.42	493.1493	493.1501	1.4	327.08736, 163.0394; 133.01353 119.0494
**28**	254, 361	Quercetin*	C_15_H_9_O_7_^−^	14.27	301.0314	301.0315	0.3	179.0502, 178.9986, 177.0193, 151.0036; 107.0129
**29**	254, 354	Isorhamnetin*	C_16_H_11_O_7_^−^	15.05	315.0510	315.0511	0.4	301.0353, 192.0423, 179.0502, 178.9985, 177.0193, 151.0035; 107.0129
**30**	251, 351	Rhamnetin*	C_16_H_11_O_7_^−^	18.08	315.0510	315.0500	2.1	301.0353, 300.0328, 192.0423, 179.0502, 178.998, 177.0193, 151.0036; 107.0129
**31**	206	Trihydroxyoctadecenoic acid	C_18_H_33_O_5_^−^	18.49	329.2333	329.2331	0.6	393.2282, 349.2384
**32**	206	Trihydroxyoctadecenoic acid	C_18_H_33_O_5_^−^	19.36	329.2333	329.2331	0.6	393.2282, 349.2383
**33**	–	Trihydroxyoctadienoic acid	C_18_H_31_O_5_^−^	19.55	327.2177	327.2178	0.5	391.2217
**34**	–	Sterol (Peniocerol derivative)	C_27_H_45_O_7_^−^	19.65	481.3171	481.3170	0.6	463.3165
**35**	–	Hydroxytetraoxodocosanoic acid	C_22_H_35_O_7_^−^	22.25	411.2389	411.2388	0.5	311.1684
**36**	–	Hydroxytetraoxodocosanoic acid	C_22_H_35_O_7_^−^	23.00	411.2389	411.2388	0.6	311.1685
**37**	–	Unknown	C_30_H_41_O_8_N_3_^−^	23.52	571.2888	571.2883	0.9	–
**38**	–	Sterol (Peniocerol derivative)	C_25_H_40_O_7_^−^	23.98	452.2783	452.2782	0.2	463.3168

*Compounds identified with spiking experiments with authentic standards

UHPLC-PDA-ESI-HRMS/MS, Ultra-High-Performance Liquid Chromatography-Photodiode Array Detector-Electrospray Ionization-High Resolution Tandem Mass Spectrometry.

#### Organic Acids and Other Phenolic Derivatives

Peaks 1–6 with deprotonated molecules at *m/z*: 133.01357, 191.01907, 147.02913, 205.03491, and 137.02357 were identified as malic, citric, and hydroxyglutaric acid (Schor et al., 2002) and its isomer, homo(iso)citric and hydroxybenzoic acids,(Mocan et al., 2018) respectively. Peak 7 was assigned tentatively as a dehydro-shikimic acid (C_7_H_11_O_5_^−^) while peak 8 with a parent ion at *m/z*: 175.0243 MS^2^ ions at *m/z*: 115.0071; 113.01269; and 87.00942, was identified as ascorbic acid (C_6_H_7_O_6_^−^) (Boonpangrak et al., 2016). Peak 10, 11, and 20 were tentatively identified as benzoyl aspartic acid derivatives whose molecular ions [M-H]- were at *m/z* 236.0561, 236.0562, and 262.0562 (Nakazaki et al., 1963). Finally, peak 21 was identified as nonanodioc acid and azelaic acid (187.0972, C_9_H_15_O_4_^−^) (Garelnabi et al., 2010). All these compounds were previously described exhibiting a similar fragmentation pattern and to the best of our knowledge have so far not been identified in Sancayo fruits. L-Malic acid is the naturally occurring form found in fruits as apple, blueberries, grapes, apricots, peaches, pears, and maqui except in cactus fruits. Isocitric acid is an organic acid very common in fruits and vegetables which is mainly found at high concentrations in citrus fruits (El-Mostafa et al., 2014; Aragona et al., 2018).

#### Hydroxycinnamic Acids

Peak 9 was identified as caffeoyl-*O*-hexoside (C_15_H_17_O_9_^−^), while two compounds (peak 12 and 13) had similar deprotonated molecules [M-H]- at *m/z* 353.0513. MS/MS fragmentation pattern of peak 12 (*m/z* 191.0191; 173.0085; 154.9978; 111.0078) indicated the presence of caffeoyl-(iso) citric acid while peak 13 was identified as its isomer (Masike et al., 2017). In similar manners, peaks 14 and 16 with ions [M-H]- at *m/z* 337.0564 and 337.0563 were identified as two coumaroyl-(iso) citric acids (C_15_H_13_O_10_^−^) (Masike et al., 2017). Peak 19 was detected at 11.90 min and identified as feruloyl-(iso) citric acid based on its deprotonated molecule [M-H]- at *m/z* 367.0671 (C_16_H_15_O_10_^−^), diagnostic MS/MS fragmentations (193.0506; 179.0344; 163.0395; 111.0079), and UV data (227; 288 and 327 nm) (Masike et al., 2017). Peaks 22 and 23 were identified as isomers of the derivative methylcoumaroyl(iso) citric acid (C_16_H_15_O_9_^−^), and finally peak 24 as methylferuloyl (iso) citric acid (C_17_H_17_O_10_^−^).

#### Flavonoids

Several metabolites were detected as flavonoids (UV max 254–354 nm). Thus, peak 15 with a deprotonated molecule at *m/z* 609.14508 was identified as rutin (C_27_H_29_O_16_^−^) (Mocan et al., 2018) and peak 17 with a deprotonated molecule at *m/z*: 623.16071 and MS fragments at 315.0509, 301.0353, as rhamnetin/isorhamnetin-3-*O*-rutinose (C_28_H_31_O_16_^−^) (Brito et al., 2014) or rhamnetin/isorhamnetin-*O*-neohesperidose (since isorhamnetin aglycone was found using standard). Peak 18 was identified as the flavanonol taxifolin (C_15_H_11_O_7_^−^) (dihydroquercetin) that is a bioactive flavanonol commonly found in grapes (Yang et al., 2016). Peak 28 with an anion at *m/z:* 301.03140, was assigned as quercetin (C_15_H_9_O_7_^−^) using authentic standard, and peaks 29 and 30 as two isomers of methyl quercetin (C_18_H_33_O_5_^−^), producing flavone diagnostic fragments at *m/z*: 301.0353, 179.0502, and 151.0035; 107.0129 isorhamnetin and rhamnetin, respectively (El-Mostafa et al., 2014; Aragona et al., 2018). Spiking experiments using some available standards (rutin, quercetin, rhamnetin, and isorhamnetin) were performed.

#### Fatty Acids

Peaks 35 and 36 with ions at *m/z*: 411.23883 were tentatively identified as isomers of the fatty acid pentahydroxy-oxodocosanoic acid (C_22_H_35_O_7_^−^) while peaks 31 and 32 with ions at *m/z*: 329.2331 and 329.2330 were tentatively identified as trihydroxy-octadecenoic acid and its positional isomer (C_18_H_33_O_5_^−^) (Toomik et al., 2012).

#### Sterols

Peak 34 at *m/z*: 481.3171 was tentatively identified as peniocerol derivative (C_27_H_45_O_7_^−^), while peak 38 as its demethylated derivative (C_25_H_40_O_7_^−^). Peniocerol is a sterol isolated and reported from *Myrtillocactus geometrizans* (Mart. ex Pfeiff.) Console (Cactaceae) (Céspedes et al., 2005).

#### Unknown

Peak 25, 26, and 27 with parent ions at *m/z:* 493.1493, 327.08740, and 493.1493 were unknown compounds tentatively identified as hydroxycinnamic acid derivatives (163.0938, C_9_H_7_O_3_^−^) possibly isoamericanol B1, C1 derivatives (CAS 77879-90) a constituent of *Phytolacca dioica* L., a dioxin which has the coumaroyl moiety in its structure (Di Petrillo et al., 2019).

### Biological Activity

#### Antioxidant Activity

DPPH, FRAP, and ABTS (TEAC) tests were used to measure the antioxidant activity of *C. brevistylus* fruits for the first time (see **Table 2**). These capacities were compared to other fruits previously reported by us including other South American fruits (Céspedes et al., 2010; Ramirez et al., 2014; Simirgiotis et al., 2016; Guerrero-Castillo et al., 2019). In addition, three main types of metabolites were tentatively identified in this work, organic acids, hydroxycinnamic acids, and flavonoids, besides polyunsaturated fatty acids (**Table 1**), that could be responsible for the antioxidant power. However, the ascorbic acid content of *C. brevistylus* fruits (38.46 ± 3.72 mg/100 g) was higher than that reported for another south American Cactaceae: Copao pears (*Eulychnia acida* Phil.), 35.7 mg/100 g (Jiménez-Aspee et al., 2014). Furthermore, the total phenolic values of *C. brevistylus* fruits, TPC: 24.34 ± 3.67 mg GAE/g dry weight, (**Table 2**) together with total flavonoids (TPC: 13.33 ± 1.88 mg quercetin/g dry weight, **Table 2**) were higher to those published for the *Opuntia ficus-indica* (L.) Mill. pears (from 9.64 mg GAE/g dry weight for the red variety to 12.28 mg GAE/g dry weight for the green variety and flavonoids: 2.45 to 3.07 mg Q/g dry weight, for the same varieties, respectively (El-Mostafa et al., 2014), and were higher to those reported for Copao pears, (47.42 mg GAE/100 g MeOH extract and 0.10 mg quercetin/100 g MeOH extract (Jiménez-Aspee et al., 2014); also the TPC was half to that the Chilean guava fruits *Ugni molinae* Turcz., (50 mg GAE/g dry weight) which is situated in the average of antioxidant edible fruits (Balasundram et al., 2006; Pinto et al., 2009; Rodríguez-Roque et al., 2014). Also, the TPC values were half lower to those measured for Chilean blueberries (45.86 ± 3.46 mg GAE/g) (Ramirez et al., 2015) and TFC nearly half to that of the *Luma apiculata* (DC.) Burret berries (29 mg Q/g) (Simirgiotis et al., 2013). In the DPPH assay, *C. brevistylus* fruit extract (47.45 ± 0.23 μg/mL) was closer to that published for lemon fruits cultivated in Northern Chile (Brito et al., 2014) and Copao pears (IC_50_ 37.4 μg/mL, from the Elqui valley (Jiménez-Aspee et al., 2014). The ABTS values (IC_50_ = 225.12 μM TE/g dry weight) were closer to maqui berries, *Aristotelia chilensis* (Molina) Stuntz berries (254.8 μM TE/g dry weight: 63.79 μg/mL) and Açai berries, *Euterpe oleraceae* Mart. (208.7 μM TE/g dry weight: 52.23 μg/mL) (Gironés-Vilaplana et al., 2014), while the FRAP values (155.34 ± 3.67 μM TE/g dry weight) where closer than those of Acai (157.9 μM TE/g dry weight) and lower than maqui berries (254.2 μM TE/g dry weight). These data can classify *C. brevistylus* fruit extracts with moderate to high antioxidant activity, such as plum, cherries, and strawberries (Gironés-Vilaplana et al., 2014; Ramirez et al., 2015; Simirgiotis et al., 2016).

**Table 2:** DPPH assay, ABTS antioxidant activity (ABTS), ferric reducing antioxidant activity (FRAP), total phenolic content (TPC), total flavonoid content (TFC), and ascorbic acid content (AA) of *Corryocactus brevistylus* edible fruit extract (*n*= 5).

Species	DPPH^−a^	ABTS^b^	FRAP^b^	TPC^c^	TFC^d^	AA^e^
*Sanky pulp*	47.45 ± 0.23	225.12 ± 2.42	155.34 ± 3.67	24.34 ± 3.67	13.33 ± 1.88	38.46 ± 3.72
Gallic acid^*^	5.47 ± 0.25	–	–	–	–	–
Quercetin^*^	7.08 ± 0.65	–	–	–	–	–

^a^Antiradical DPPH and ABTS activities are expressed as IC50 in µg/mL for extracts and compounds; ^b^Expressed as μM TE/g dry weight. ^c^TPC expressed as mg gallic acid/g dry weight; ^d^Total flavonoid content (TFC) expressed as mg quercetin/g dry weight; ^e^Expressed as mg per 100 g. *Used as standard antioxidants. Values in the same column are significantly different (at p < 0.05).

AA, ascorbic acid content; FRAP, ferric reducing antioxidant power test; TPC, total phenolic content; TFC, total flavonoid content.

#### Gastroprotective Activity

The effects of the CEX on the model of HCl/EtOH in mice are presented in **Table 3**. An oral administration of CEX at 10, 25, 50, and 100 mg/kg inhibited the formation of gastric lesions compared with control group (P < 0.01). It is also clear that the inhibition by CEX did not display a dose-response relationship. The inhibition displayed by CEX at 50 and 100 mg/kg, p.o. (63% and 73%) was similar to that obtained with lansoprazole (67%). In the second experiment, we decided to evaluate the possible mechanism of gastroprotective action of CEX at a single oral dose of 50 mg/kg. **Table 4** shows the results of CEX on the gastric lesions induced by HCl/EtOH in mice pretreated with indometacin (IND, 10 mg/kg, s.c.), NEM (10 mg/kg, s.c.), or L-NAME (70 mg/kg, i.p.).

**Table 3:** Gastroprotective activity of CEX on HCl/EtOH-induced gastric lesions in mice.

Compound	n	Lesion index (mm)	% Lesion reduction	Dose (mg/kg)
CEX-10	7	36.6 ± 4.7^*^	18^**^	10
CEX-25	7	29.7 ± 3.3^*^	34^**^	25
CEX-50	7	16.7 ± 3.9^*^	63	50
CEX-100	7	12.0 ± 3.4^*^	73	100
Lansoprazole	7	15.7 ± 2.9^*^	67	30
Control	7	45.3 ± 5.3^**^	–	–

The results are expressed as mean ± SD. *P < 0.01 compared with the control and **P < 0.01 compared with lansoprazole (ANOVA followed by Dunnett’s test). n = number of mice.

**Table 4:** Effect of crude ethanolic extract (CEX) on the appearance of gastric lesions induced by HCl/EtOH (p.o.) in Indomethacin- (IND), *N*-ethylmaleimide- (NEM), and *N*-nitro-L-arginine methyl ester- (L-NAME) pretreated mice.

Treatment	Dose (mg/kg)	Lesion index (mm)
Control	–	41.6 ± 4.9
IND	10	36.0 ± 3.8
NEM	10	38.6 ± 4.4
L-NAME	70	40.7 ± 5.1
**CEX**	50	16.7 ± 3.5*
IND + **CEX**	10 + 50	35.9 ± 2.8
NEM + **CEX**	10 + 50	37.7 ± 5.3
L-NAME + **CEX**	70 + 50	18.0 ± 3.1*
Carbenoxolone	100	14.6 ± 4.2*

Results are expressed as mean ± SD, n = 7. Analysis of variance followed by Dunnett’s test. *P < 0.01 compared with the respective control.

IND, Indomethacin; NEM, N-ethylmaleimide; L-NAME, N-nitro-L-arginine methyl ester.

Regarding mode of gastroprotective action, pretreatment with NEM (an SH blocker) reduced the gastroprotective activity of CEX, suggesting that the protective effect of this extract is through the participation of endogenous SHs (**Table 4**). Regarding prostaglandins, they seem to have participation in the gastroprotective action of CEX since the inhibition was reduced by pretreatment with IND (**Table 4**).

## Discussion

Today, it is well known that gastric diseases are a problem around the world and are commonly associated with the consumption of non-steroidal anti-inflammatory drugs (Wallace, 2019a). Despite the wide use of therapies toward gastric ulcers, the recurrence index is high. In this sense, gastroprotective natural drugs could be a cheap and affordable alternative to prevent gastric ulcers and improve the way of life of humans living with this gastrointestinal condition. The disability of the balance between aggressive and defensive factors in the gastric mucosa could lead to gastric ulcers (Lewis and Hanson, 1991). Several medicinal plants and fruits showed ability to protect the gastric mucosa in different animal models (Lewis and Hanson, 1991; Tundis et al., 2008; De Lira Mota et al., 2009; Khan et al., 2018). Plants have produced interesting gastroprotective drugs such as carbenoxolone from *Glycyrrhiza glabra* L., gefarnate from cabbage, solon from sophoradin, to mention a few (Lewis and Hanson, 1991). Furthermore, Cactaceae plants have demonstrated gastroprotective activity. As an example, the medicinal plant *O. ficus-indica* (Cactaceae) has showed gastroprotective, hepatoprotective, neuroprotective, and antioxidant effects, it has also showed antimicrobial and nutraceutical properties, the extract could serve as diuretic, anti-cancer, anti-stress, anti-diabetic and anti-inflammatory and is good for the healing of skin lesions (El-Mostafa et al., 2014; Aragona et al., 2018; Aruwa et al., 2019; Smeriglio et al., 2019).

### Mode of Gastroprotective Action

Peptic ulcer disease (PUD) is formed when the protective mechanisms of the gastrointestinal mucosa, such as bicarbonate secretion and mucus, are surpassed by the damaging effects of pepsin and gastric acid (Sung et al., 2009). The main factors involved in the development of ulcers include an increase in gastric acid secretion and a decrease in mucosal protection due to the reduction of mucus secretion, mucosal blood flow, and prostaglandin biosynthesis (Konturek et al., 1990, Brzozowski et al., 2005). Constant renewal of epithelial cells is fundamental in the protection of the mucosa of the gastrointestinal tract. The most important targets to study the mechanism of action of this pathology are NO, endogenous sulfhydryls, and prostaglandin biosynthesis. Nitric oxide in the gastrointestinal tract play a fundamental role in the correct repairing of the gastric mucosa (Wallace and Ma, 2001, Wallace, 2019b). Indeed, it has been demonstrated that endogenous NO regulates the gastric mucosal blood flow, angiogenesis, and gastric mucus secretion. In this study, pretreatment with L-NAME have not attenuated the gastroprotection of CEX extract (**Table 4**). This finding suggests that endogenous NO have no participation in the gastroprotective activity of this extract. Endogenous PGs are involved in the mechanism of gastroprotective action elicited by mild irritants and necrotizing agents. PGs stimulate release of mucus and bicarbonate, inhibit the gastric acid secretion, and increase blood flow on gastric mucosal (Wallace, 2019a). In our case, PGs seem to have participation in the gastroprotective action of CEX since the inhibition was reduced by pretreatment with IND.

Endogenous sulfhydryls such as glutathione are well-known agents whose main function is the protection of the gastric mucosa. Glutathione protects the integrity and permeability of the cell membrane and may act as antioxidant as a scavenger of free radicals; it works in the maintenance of immune function, regulation of protein synthesis and degradation, and the maintenance of global protein structure (Lewis and Hanson, 1991). In this study, pretreatment with NEM (an SH blocker) reduced the gastroprotective effect of CEX, suggesting that the protective effect of this extract is can be explained thorough the participation of endogenous SHs.

### Infections

The mortality of PUD decreased in the last few decades, due to treatment of infections with the bacteria *H. pylori* (Lau et al., 2011). The significance of several bioactive compounds against this bacteria such as: polysaccharides obtained from fruits (acidic heteroxylans, from *Olea* fruits, and *Maytenus ilicifolia* Mart. ex Reissek leaves), arabinogalactanes (mango, jambo, and lyceum fruits), rhamnogalacturonanes (grapes and ginseng), terpenoids (such as limonene, pinene, citral, lupeol, ursolic acid, and nomiline), and flavonoids such as quercetin (Bonifácio et al., 2014) have been discussed (Khan et al., 2018). These compounds can difficult the adherence, colonization, and invasion of *H. pylori* into the gastric cell wall and prevent gastric cancer formation, suppressing cancer growth, which is prevalent in *H. pylori* infected patients.

### Fruit Extracts and Gastroprotection.

A comparative analysis with gastroprotective extracts from fruits are explained below. The berries from *Morus nigra* L. and *Rubus niveus* Thunb showed antioxidant and gastroprotective activities due to the presence of flavonoids. The extracts from these species inhibited gastric lesions at 300 mg/kg by 64.06% and 81.86% respectively. The authors indicated that the studied berries are a source of antiulcer compounds and this may be related to their high polyphenol contents (Nesello et al., 2017). In our case, the inhibition displayed by CEX at 100 mg/kg, p.o. was 73% considering a lower dose. Previous study reported that the ethanolic extracts from seeds of *Pouteria campechiana* (Kunth) Baehni at 100 mg/kg (80% inhibition) and 200 mg/kg (90% inhibition) showed gastroprotective effect in the ethanol-induced ulcer model in rats (Elsayed et al., 2016). In the same study the authors published the isolation of the phenolic compounds: protocatechuic acid, gallic acid, quercetin, myricetin, myricetin-3-*O*-L-rhamnoside, and myricetin-3-*O*-D-galactoside, which were linked to the antiulcerogenic effects (Elsayed et al., 2016). In our case, the inhibition displayed by CEX at 100 mg/kg was close to that observed with *Pouteria* extracts. Schlickmann et al., 2015 showed that the methanolic extract from seeds of *Mimusops balata* fruits had more gastroprotective activity than peel or pulp at 300 mg/kg. This activity was associated to the maintenance of Glutathione (GSH) levels, reduction of Lipid Peroxidation (LPO) content, inhibition of neutrophil migration, and potent free radical scavenger activity (Schlickmann et al., 2015). Then, seed extract was subjected to chromatographic analyses and the flavonoid taxifolin was isolated, that also displayed gastroprotective effect on HCl/EtOH at 1.14 mg/kg (Schlickmann et al., 2015). Interestingly, *C. brevistylus* pear fruits has the presence of several dietary phenolic compounds and other important dietary compounds such as antioxidant fatty acids, that could prove its ethnobotanical and medicinal use in Perú. As an example, we demonstrated that Sanky fruits belonging to the Cactaceae family has important flavonoids such as rutin, isorhamnetin-3-*O*-rutinose, taxifolin, quercetin, and methyl quercetin in a similar way to the *O. ficus-indica* (L.) Mill. (Cactaceae). These compounds have been reported to reduce gastric ulcer formation (Suzuki et al., 1998; De Lira Mota et al., 2009). Therefore, the presence of those dietary flavonoids could explain in part the antioxidant and gastroprotective activities.

## Conclusions

From the edible endemic *Corryocactus brevistylus* (Sanky) pears, 38 compounds were detected by UHPLC-ESI-HR-MS including twelve organic acids (peaks 1–8, 10–11, and 20, 21), nine hydroxycinnamic acids (peaks 9, 12–14, 16, 19, and 22–24), three isoamericanol derivatives (25–27), six flavonoids (peaks 15, 17, 18, 28–30), five fatty acids (peaks 31–33 and 35–36), and two sterols (peak 34 and 38) for the first time. The CEX from *C. brevistylus* fruits had antioxidant (DPPH, ABTS, and FRAP) and gastroprotective activities on the model of HCl/EtOH-induced gastric lesions in mice. Our results suggest that sulfhydryl groups, prostaglandins, and antioxidant phenolics are involved in the mode of gastroprotective action of this edible cactus pears. Finally, this study proves that these endemic fruits have a nutritional added value supported by the detection of several dietary phenolic compounds and its consumption is recommended for the presence of those bioactive components with reported gastroprotective and antioxidant activities. Bioassay-guided fractionation and further isolation of main compounds from this endemic fruit is needed to determine the main bioactive metabolites.

## Data Availability Statement

The datasets generated for this study are available on request to the corresponding authors.

## Ethics Statement

The animal study was reviewed and approved by The Animal Use and Care Committee of the Universidad de Chile.

## Author Contributions

BS, TC, and CA designed this study. FC and JT collected the edible fruit. CC and TC performed the experiments. MH and TC performed extraction process and biological activities, while JB and BS analyzed the LC/MS data. Draft preparation: JE, MS, CA, and BS. Funding acquisition: BS. All authors read and approved the final manuscript.

## Funding

Financial support came from FONDECYT REGULAR N° 1170871. MS acknowledge FONDECYT 1180059, JE is grateful for support from FONDECYT project N° 11160877 and CONICYT PAI/ACADEMIA project N° 79160109.

## Conflict of Interest

The authors declare that the research was conducted in the absence of any commercial or financial relationships that could be construed as a potential conflict of interest.
